# Dissecting the Phospho-Regulatory Landscape of Protein Kinase N1 (PKN1) and Its Downstream Signaling: Functional Insights into the Activity-Dependent and Disease-Relevant Phosphosites

**DOI:** 10.3390/ijms27052137

**Published:** 2026-02-25

**Authors:** Sreeshma Ravindran Kammarambath, Leona Dcunha, Athira Perunelly Gopalakrishnan, Yashi Shailendra Gautam, Furqaan Ahmed Basha, Prathik Basthikoppa Shivamurthy, Inamul Hasan Madar, Rajesh Raju

**Affiliations:** Centre for Integrative Omics Data Science (CIODS), Yenepoya (Deemed to be University), Mangalore 575018, Karnataka, India; sreeshmarkk@gmail.com (S.R.K.); leonadcunha@gmail.com (L.D.); 19228@yenepoya.edu.in (A.P.G.); yashigautam2000@gmail.com (Y.S.G.); furqaanahmed29@gmail.com (F.A.B.); prathikbsgowda@gmail.com (P.B.S.)

**Keywords:** PKN1, phosphoproteomic, cytoskeletal regulation, insulin signaling, metabolic pathways, HCC

## Abstract

Protein Kinase N1 (PKN1) is a PKC-related serine/threonine kinase of the AGC group within the eukaryotic protein kinase superfamily (ePK) that orchestrates oncogenic, metabolic, and cytoskeletal signaling. Despite these critical roles, the phosphorylation-dependent regulatory network of PKN1 remains largely undefined. We performed a large-scale phosphoproteomic data integration of publicly available human datasets (892 profiling datasets and 191 differential datasets) to identify recurrent PKN1 phosphorylation sites. This analysis identified two predominant PKN1 phosphosites, S562 and S916, that were frequently observed and differentially regulated across studies. The S916 maps to a turn motif (TM) in the AGC group of kinases, which is evolutionarily conserved among PKN paralogs, while S562 is non-conserved and appears to be PKN1-specific. Co-regulation and enrichment analyses suggest that S916 is associated with insulin/AMPK signaling and metabolic pathways, whereas S562 co-occurs with phosphosites involved in cell division, cytoskeletal regulation, and microtubule cytoskeleton organization. Integrating predicted and experimentally validated kinases, substrates, and interactors, we reconstructed a phospho-regulatory network that positions PKN1 at the crossroads of cytoskeleton organization and metabolic signaling. To assess the disease relevance of these phosphorylation events, we integrated transcriptomic and phosphoproteomic data from the hepatocellular carcinoma database (HCCDB). PKN1 was markedly up-regulated in HCC, and its phosphorylation at S916 was positively co-regulated with multiple oncogenic and proliferation-associated protein phosphosites. These results predict S562 and S916 as potential sites for targeted biochemical validation and functional experiments. The identification of S562 and S916 as key regulatory sites provides new mechanistic insight into PKN1 activation and highlights potential avenues for therapeutic targeting.

## 1. Introduction

PKN1, or Protein Kinase N1, also referred to as Protein Kinase C-related kinase 1 (PRK1), is a serine/threonine kinase composed of 942 amino acids, encoded by the PKN1gene located on 19p13.12 in humans [1,2]. PKN1 is mainly localized in the cytosol and can associate with the plasma membrane and cytoskeletal components [3]. PKNs are PKC-related serine/threonine kinases of the AGC group within the eukaryotic protein kinase superfamily (ePK) and comprise PKN1, PKN2, and PKN3 [2]. All three PKNs share similar domain structures with a conserved kinase domain in the C-terminal and N-terminal HR1 domains that mediate Rho GTPase binding [4]. The HR1 regions and the kinase domain in PKN1 are found to be conserved along human, rat, mouse, and other vertebrates [5]. Currently, T774 is considered an autophosphorylation site of PKN1 within the kinase domain, which is critical for its activation [6]. Moreover, phosphorylation at S377, S561, and T778 have been linked to PKN1 enzyme activity and regulation [7]. Conserved serine/threonine residues within their regulatory domains represent critical phosphorylation sites for upstream kinases such as PDK1 [8,9]. Phosphorylation and activation of PKN1 by Rho GTPases and PDK1 in response to cellular stress, growth factors, and mechanical stimuli are associated with cytoskeletal reorganization and are proposed as an intermediate that links Rho signaling to actin dynamics and stress-activated protein kinase pathways [3]. The PKN1 mutation or knockdown consequently suppresses the Rho-mediated actin remodeling, impairs the cell migration, and increases sensitivity to mechanical stress [10]. In response to the Rho signal, PKN1 phosphorylates the cytoskeletal proteins that regulate actin filament assembly and maintain cell shape, thereby supporting proper cell motility and adhesion. Supporting this, PKN1 depletion reduces the density of stress fibers and focal adhesions during cell migration [10,11]. As a central effector of the Rho signaling pathway, PKN1 has been implicated in the phosphorylation and activation of downstream targets like MARCKS in mammals. Regulation of the Rho/ROCK pathway [12,13] is also associated with the regulation of MAPK signaling [3]. The Rho signaling cascade is implicated in human cancers, and PKNs have emerged as potential therapeutic targets due to their key regulatory functions [11,14].

With a significant role in cytoskeletal dynamics, cell migration, and signal transduction, dysregulation of PKN1 is associated with cancer progression and metastatic diseases [15,16]. Chromosome 19 alterations, potentially involving PKN1, have been linked to tumorigenesis and abnormal cell growth [17]. Further, several sequence variations or altered expressions of PKN1 have been associated with cancer and inflammatory disorders [14,15,16,18,19]. The phosphorylation of PKN1 at T774 has been associated with abnormal signaling events in prostate cancer and several other malignancies. In a recent development, a small-molecule inhibitor of PKN1 has been reported to reduce the phosphorylation of multiple sites (S377/S561 and T778/T780) in cell-based models, suggesting a potential role in modulating the cancer-related signaling pathways [6]. The Rho-associated coiled-coil kinase (ROCK) is reported to interact with PKN1 and promote phosphorylation-dependent interactions with cytoskeletal targets for actin remodeling. PKN1/2 complexes with RhoA, RhoC, and alpha-actinin are associated with cytoskeletal dynamics in cell motility [20]. Recently, Lachmann et al. (2011) [10] identified that mutations that abolish PKN1’s catalytic activity interfere with Rho GTPases and impair stress fiber formation. Autophosphorylation at T774 of PKN1 is found to be associated with kinase activity, and related residues have been proposed to function as a molecular switch in this regulatory process [10].

Despite being a cytosolic and membrane-associated AGC group of serine/threonine kinase that regulates actin–cytoskeleton dynamics and cell migration, PKN1 (PRK1) remains incompletely characterized with respect to its phospho-regulatory network, substrates, upstream kinases, and interacting partners [15]. PDK1 (PDPK1) phosphorylates PKN1 on the activation-loop threonine (T774) and is required for insulin-stimulated actin cytoskeleton reorganization, thereby linking PKN1 to PI3K/insulin signaling [21]. The C-terminal turn-motif residue S916 is phosphorylated in a TORC2/mTOR-sensitive manner and contributes to PKN kinase activity, while mitotic phosphorylation at multiple sites (including S562 and S916) by CDK1 has been reported during drug-induced mitotic arrest; these mitotic phosphorylations promote anchorage-independent growth and migration in cell models [22,23]. Motivated by these observations and the remaining gaps in site-level regulatory detail, we performed a systematic analysis of large-scale human phosphoproteomic datasets to identify predominant and differentially regulated PKN1 phosphosites and to infer their likely upstream kinases, substrate partners, and pathway associations. Here, we systematically collected and re-analyzed publicly available human phosphoproteomic datasets to (i) identify recurrent PKN1 phosphorylation sites, (ii) infer co-regulated phosphosite networks and candidate upstream kinases, substrates, and interactors, and (iii) map functional pathway associations. This integrative approach reveals two predominant PKN1 phosphosites, S562 and S916, and generates testable hypotheses regarding their roles in mitotic control and metabolic signaling.

## 2. Results

### 2.1. Compilation of the Phosphoproteomics Datasets of PKN1 and the Identification of the Predominant Phosphosites

**Figure 1:**
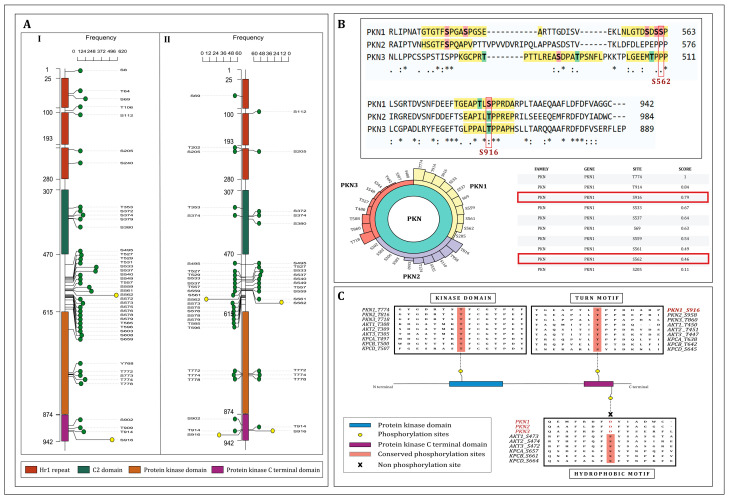
(**A**) Lollipop plot visualization of PKN1 phosphosites across (I) qualitative profiling and (II) quantitative differential phosphoproteomic datasets. (**B**) Sequence alignment showing the conservation of predominant phosphosites across the PKN family. The yellow highlighted area is the ±5 amino acid window surrounding each phosphorylation sites, serine and threonine phosphorylation sites are indicated in pink and green, respectively. In the sequence alignment, an asterisk (*) denotes complete conservation, a colon (:) indicates strong similarity between residue groups, and a period (.) denotes weak similarity (PxKD- (https://ciods.in/pxkd, accessed on 23 December 2025)). (**C**) Comparative alignment of PKN1 with representative AGC group of kinases highlighting the conserved turn-motif and hydrophobic-motif regions, emphasizing evolutionary conservation of S916 within the AGC group of kinases.

We identified 66 and 32 phosphosites of PKN1 across 892 profiling datasets and 191 differential datasets by curating over 3825 publicly available phosphoproteomic-based datasets with class 1 phosphorylation sites under various experimental conditions. These findings from the mass-spectrometry-based phosphoproteome profiling provide valuable insights that facilitate the identification of novel and previously unexplored phosphosites with potential regulatory significance in particular cellular processes. All identified PKN1 phosphosites were systematically ranked based on their detection frequency across the profiling and differentially regulated datasets (Supplementary Table S1). A Lollipop plot visualization of phosphosites within PKN1 with their detection frequencies in the datasets is illustrated in Figure 1A. Among the identified PKN1 phosphosites, S562 and S916 were the most recurrently detected and predominantly represented phosphosites, with the highest frequencies of 103 and 64 in differential regulation datasets, respectively.

Gopalakrishnan et al. (2025) have developed PxKD (https://ciods.in/pxkd, accessed on 23 December 2025 [24]), a resource that classifies kinases based on predominantly detected phosphosite annotations and their relevance to the human kinase regulatory network. Based on this classification, PKN1 is categorized as PoKD, a kinase with its predominant phosphosite outside its kinase domain [24]. The predominant phosphosites S562 and S916 are located outside the kinase domain of PKN1, whereas S916 is found within the protein kinase C terminal domain (Pkinase_C). PKN1 is phosphorylated at residues S562 and S916 by upstream kinase CDK1 under conditions of drug-induced mitotic arrest in both in vitro and cellular models. These phosphorylation events are associated with anchorage-independent proliferation and migratory behaviors in ovarian and cervical cancer cells [22]. Mutation within PKN1 (S916A) causes a substantial reduction in kinase activity compared to their respective wild-type (WT) forms [23]. Notably, despite these findings, there are currently no site-specific quantitative biochemical studies that directly associate phosphorylation at S562 or S916 to modulation of PKN1 phosphotransferase activity.

### 2.2. Conserved Phosphorylation Patterns Across the PKN Family Members: PKN1, PKN2, and PKN3

In mammals, the PKN (Protein Kinase N) family consists of three members: PKN1, PKN2, and PKN3 [3]. Although these three proteins share a high degree of sequence similarity, they have distinct regulatory properties, patterns of tissue expression, and functional roles. The PKN family are involved in diverse biological processes, including the regulation of cell cycle [25], vesicle transport [7], receptor trafficking [26], and programmed cell death [27]. As downstream mediators of Rho GTPase effectors, PKNs contribute to cytoskeletal structure, cell adhesion, proliferation, and movement. Furthermore, activation of PKN signaling pathways has been implicated in promoting cell survival across multiple tumor cell types, including prostate and breast cancers [28].

In all three PKNs, the C-terminal region encodes a serine/threonine kinase domain that shares significant structural similarity with the catalytic domain of PKC family proteins [28]. As S562 and S916 emerged as the predominant phosphosites of PKN1 in the global phosphoproteomics datasets and given that S916 is positioned within the protein kinase C terminal domain, we investigated the evolutionary conservation of these sites across the PKN family. The evolutionary phosphosite conservation across and within kinase families was assessed using the kinase-family-based conservation analysis given in PxKD (https://ciods.in/pxkd). Phosphosites S916 of PKN1, T958 of PKN2, and T860 of PKN3 were found to be conserved and reside within the protein kinase C terminal domain of the PKN family, with a score of 0.79, indicative of high conservation (threshold > 0.75) [24]. In contrast, S562 showed a lower conservation score of 0.46, reflecting limited conservation within the PKN family and highlighting its specificity to PKN1 (Figure 1B).

### 2.3. Phospho-Regulatory Mechanisms in AGC Group of Kinases: Insights into Conserved Activation Sites of PKN1

The AGC group of kinases comprises 63 evolutionarily related serine/threonine kinases, including PDK1, MRCK, AKT, PKN, PKC, SGK, S6K, MAST, ROCK, DMPK, PKA, LATS, PKG, YANK, CRIK, MSK, NDR, RSK, and GRK, which act as downstream effectors in diverse signaling pathways [29]. This AGC group of kinases shares a conserved activation mechanism that involves three significant conserved phosphorylation events: the first in the kinase domain activation loop, the second in the hydrophobic motif (HM) of the C-terminal tail, and the third in the turn motif (TM), within the protein kinase C-terminal domain [30]. A short C-terminal sequence of PKN shares similarity with the turn motif (TM), where it serves a crucial role in enzyme stabilization, and is also found to be present in other AGC groups of kinases, including PKC and AKT [31]. The phosphorylation at the TM contributes to increased kinase activity and structural stability by facilitating interactions involving the N-lobe of the catalytic core. The C-terminal domain of PKNs exhibits 40–50% sequence similarity with those of PKC and AKT and possesses a conserved sequence resembling the TM. It is already established that phosphorylation in the TM of PKN is essential for its catalytic function to phosphorylate multiple substrates. Notably, mutations within PKN1 (S916A) and PKN2 (T958A) cause a substantial reduction in kinase activity compared to their respective wild-type (WT) forms [23]. In the case of PKN3, kinase activity has been shown to depend on phosphorylation at T860 within the TM [14]. Collectively, these findings suggest that phosphorylation within these motifs is associated with the activation of PKN kinase, indicating that these post-translational modifications enhance its kinase activity. Multiple sequence alignment (MSA) of the PKN, AKT, and PKC kinase families revealed conservation at two phosphorylation sites, S774 located within the kinase domain and S916 in the TM of PKN1. These sites were conserved across all three families, suggesting functional relevance. In contrast, HM, a known regulatory site in the AGC group of kinases, was not conserved or clearly identified in PKNs. This indicates that the HM phosphorylation event is not essential for PKN activity (Figure 1C). S916 is found to be one of the predominant phosphosites of PKN1, which is in the TM. Notably, serine 562 (S562) of PKN1, which is not conserved among other kinases, represents one of the most highly phosphorylated residues within PKN1. To delineate the phospho-regulatory network of PKN1, we aimed to investigate the functional significance of its two major phosphorylation sites, S562 and S916.

### 2.4. Consistent Co-Regulation Pattern of Predominant PKN1 Phosphosites with Phosphorylation Sites in Other Proteins (POp)

To explore the potential functional association in the co-regulation pattern of PKN1, we observed phosphosites with phosphorylation sites in other proteins (POp), which are consistently co-regulated with the PKN1 predominant phosphosites, to explore their potential functional associations. To ensure the accuracy and biological relevance of the identified associations, stringent inclusion criteria were applied to the phosphosite pairs derived from Fisher’s exact test (FET). Phosphosites were retained only if they satisfied a detection frequency of at least 10% relative to the top differential frequency of the predominant PKN1 site for either positive or negative co-regulation, a statistically significant FET *p*-value below <0.05, consistent evidence of co-regulation reported in a minimum of three independent studies (PubMed IDs), and observation in at least three distinct experimental conditions (experimental code counts).

**Figure 2:**
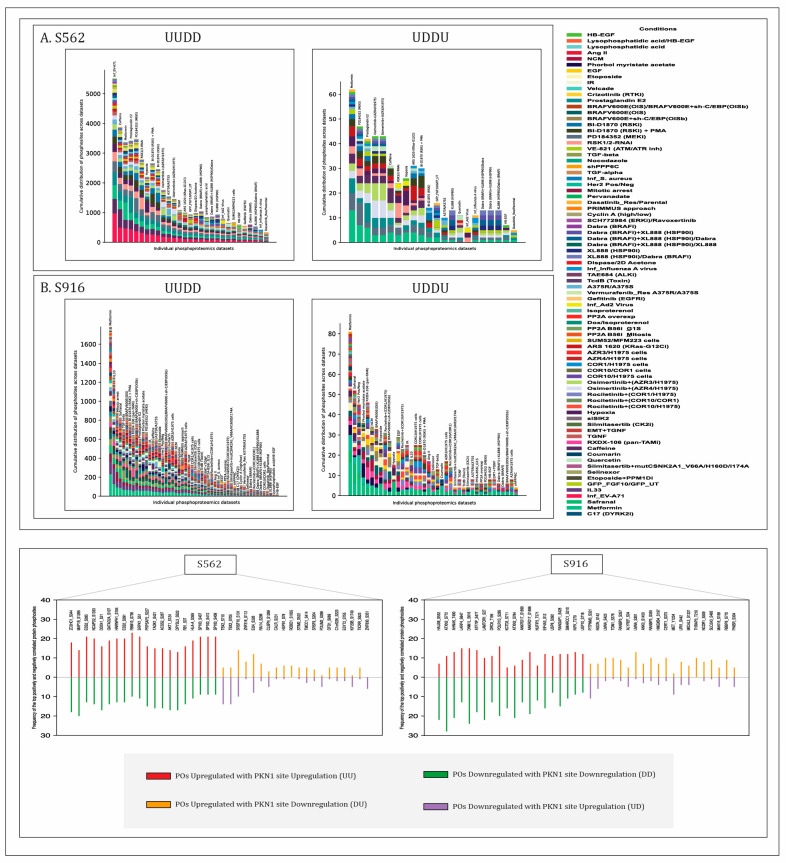
The cumulative distribution of protein phosphosites (with FET *p*-value < 0.05) across multiple datasets, representing both the positive and negative co-regulation of PKN1-specific predominant phosphosites and the co-regulation pattern of the top 20 high-confidence positively and negatively co-regulated POp of associated with PKN1 predominant phosphosites.

Analysis revealed 300 and 11 high-confidence positively and 1477 and 10 negatively co-differentially regulated POp with S562 and S916 of PKN1, respectively (Supplementary Table S2). A total of 19 POp were identified to be positively co-regulated with both S562 and S916, whereas no overlap was observed in negatively co-regulated POp. The cumulative distribution of phosphosites across multiple datasets, representing both positive and negative regulation of the PKN1 predominant phosphosites, and the co-regulation expression of the top 20 high-confidence positively and negatively co-regulated POp are illustrated in Figure 2. The upstream kinases, downstream substrates, binary interactors, and complex interactors of the predominant sites within this threshold were identified, and their functional associations with various biological processes were evaluated. Gene set enrichment analysis (GSEA) of the co-regulated proteins revealed distinct functional associations. Proteins co-regulated with S562 were enriched in the processes of cell division, cytoskeletal regulation, and microtubule cytoskeleton organization, whereas those co-regulated with S916 were associated with nucleocytoplasmic transport, insulin signaling, and AMPK signaling pathways.

### 2.5. Experimentally Validated and Predicted Upstream Kinases and Downstream Substrates of PKN1 and Their Co-Regulation with Predominant Sites

In this study, we examined experimentally validated and computationally predicted upstream kinases, as well as downstream substrates, that exhibit positive and negative co-regulation with the PKN1 predominant phosphosites. The analysis revealed seven upstream kinases positively co-regulated with these sites, while none were negatively co-regulated. Among these, three upstream kinases were consistent with the predictions reported by Johnson et al. (2023) [32], while five were identified through computational prediction tools discussed in the methodology. Notably, CDK2 and CSNK1A1 were predicted to be upstream kinases for S562, with CDK2 (Y14, Y15) and CSNK1A1 (T321) positively co-regulated with S562, whereas CDK13 and SRPK1 were identified as predicted upstream kinases of S916, with SRPK1 (S51) and CDK13 (T1246, S315, S317) positively co-regulated with S916.

PKN1 was found to have 30 experimentally validated downstream substrates, including 10 autophosphorylation sites of PKN1 (Phosphositeplus: [33]). None of the experimentally validated substrates exhibited co-regulation with either of the predominant PKN1 phosphosites. To further elucidate potential regulatory relationships, predicted PKN1 substrates were identified through the computational prediction tools and positional scanning peptide array (PSPA) analysis by Johnson et al. (2023) [32]. This analysis revealed 14 positively and 2 negatively co-regulated substrates with S562 and 127 positively and 2 negatively co-regulated substrates with S916 (Supplementary Table S3).

### 2.6. Downstream Substrates and Other Co-Regulated Protein Phosphosites with S562 in Cytoskeletal Regulation and S916 in Insulin Signaling Pathway

Enrichment analysis of the co-regulated proteins, along with upstream kinases and downstream substrates, revealed their functional connections to several biological pathways. Proteins co-regulated with S562 show significant enrichment in cell division, cytoskeletal regulation, and chromatin remodeling. PKN1 is known to be an effector of the Rho family small GTP-binding proteins, which are key modulators of the actin cytoskeleton, playing a role in various signaling pathways related to cell adhesion and motility [13,34]. From our analysis, ten proteins associated with Rho GTPases were identified to be positively co-regulated with S562, among which ABI2 (S242) was found to be the direct substrate of PKN1. In addition, proteins associated with cytoskeletal regulation and the cell cycle were found to be positively co-regulated with S562, further underscoring the functional relevance of this signaling axis. Positively co-regulated proteins with S916 were strongly associated with the insulin signaling pathway, including predicted substrates of PKN1- PDPK1 (S241), GFPT2 (S244), GFPT1 (S261), PPP1R3D (S25), IRS2 (S306, S577), and PRKAA1 (S496). PDPK1 (S241) was identified as the binary interactor of S916.

Park et al. (2013) reported that PKC activation causes the phosphorylation of IRS2 at S303 and blocks insulin-induced tyrosine phosphorylation of IRS2 at key sites for downstream signaling (notably Tyr911), thereby impairing the activation of the PI3K/Akt/eNOS pathway [35]. IL-4-induced tyrosine phosphorylation increases IRS2 activity, while serine phosphorylation suppresses it [36]. PDPK1 activates AKT by phosphorylation in response to insulin, leading to the phosphorylation of GSK3 and subsequent glycogen synthesis [37]. PDPK1 phosphorylation at S241, located within the activation loop of PDPK1, is essential for its catalytic activity, thereby activating downstream signaling [38,39]. GFPT1 serves as the key enzyme that initiates and regulates the rate-limiting reaction in the hexosamine biosynthetic pathway, a crucial metabolic process linking glucose flux to protein glycosylation. Notably, phosphorylation at S261 has been reported to enhance the enzymatic activity of GFPT1 [40], potentially promoting the hexosamine biosynthetic pathway. PRKAA1 encodes the catalytic α1 subunit of AMPK and regulates glucose and lipid metabolism. PKC activation inhibits PRKAA1 at S496, which is associated with insulin resistance and obesity [41].

**Figure 3:**
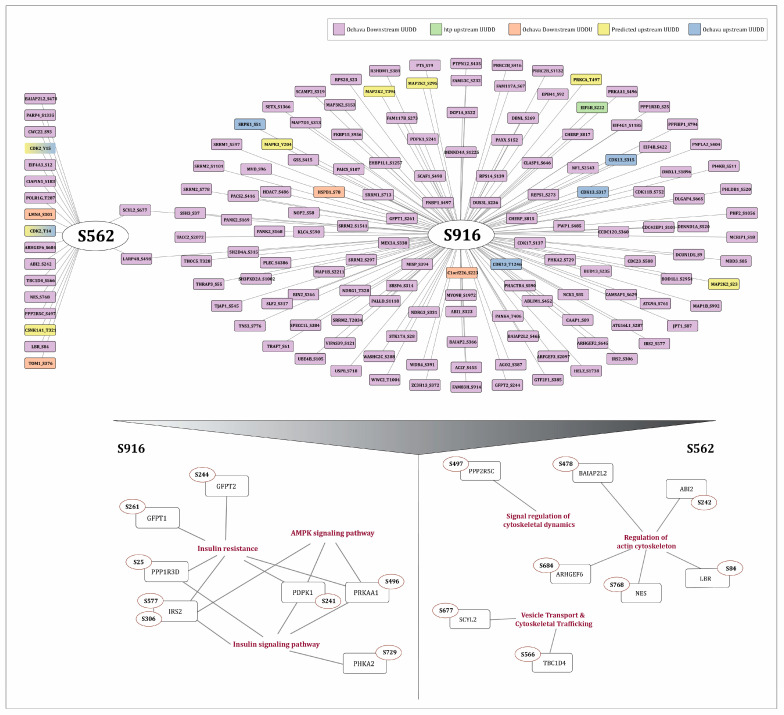
Network representation of the phosphosites of downstream substrates of PKN1 co-regulated with S562 and S916. Each color represent downstream substrates from different source and their co-regulation type, mixed colors indicating overlapping upstream regulatory inputs. Functional enrichment analysis of the network is also depicted, highlighting significant association with biological processes related to insulin signaling and cytoskeletal organization.

Collectively, these observations suggest that S916 of PKN1 is integrated with a regulatory phosphorylation network of insulin signaling by various activation and inhibitory events. This co-regulation highlights PKN1 as a potential signaling node influencing the insulin signaling pathway. The downstream substrates and their enrichment in various signaling pathways are depicted in Figure 3. However, targeted experimental validation will be essential to establish the functional relevance of these predicted interactions.

### 2.7. PKN1 Association with Metabolic Pathways: Co-Regulation of Predominant Phosphosites of PKN1 with Metabolic Enzymes

**Figure 4:**
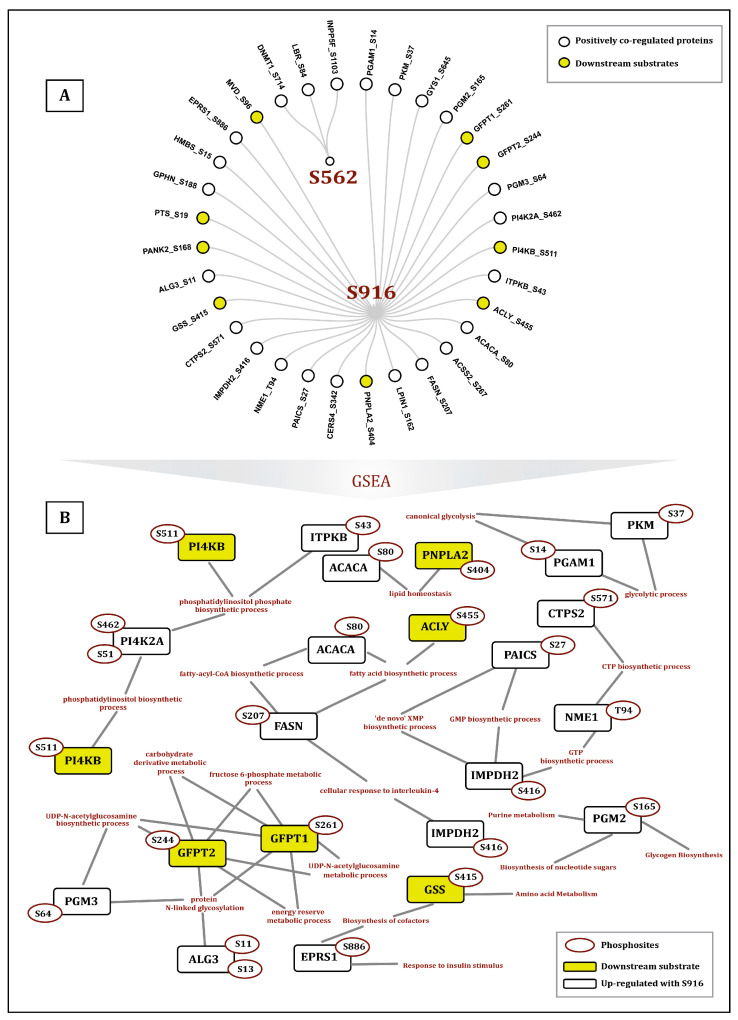
(**A**) Network visualization showing the metabolic enzymes and their phosphorylation sites that are positively co-regulated with PKN1 S562 and S916. (**B**) Functional enrichment of the metabolic enzymes, co-regulated with PKN1 S916, showing significant enrichment across multiple metabolic pathways.

PKN1 acts as an effector of Rho GTPase and plays a regulatory role in several metabolic pathways [34]. Notably, 29 metabolic enzymes associated with various metabolic pathways exhibited positive co-regulation with S916, while only 3 were positively co-regulated with S562. These enzymes are associated with several biological processes such as the glycolytic process, lipid regulation, and fatty acid biosynthetic process. Among them, PI4KB (S511), GFPT1 (S261), PANK2 (S168, S169), PNPLA2 (S404), MVD (S96), GFPT2 (S244), ACLY (S455), GSS (S415), and PTS (S19) are predicted to be direct substrates of PKN1 and positively co-regulated with S916. GFPT1 serves as a key enzyme in the hexosamine biosynthetic pathway (HBP) that provides precursors for protein and lipid glycosylation, and phosphorylation at S261 results in its increased enzymatic activity [40]. Phosphorylation of PNPLA2 at S404 has been identified as a downstream target of CAMKK2-AMPK signaling in CRPC cells [36]. AKT phosphorylates MVD at S96, which in macrophages increases the activation of RAC1, thereby enhancing macrophage survival, as mutation of MVD induces apoptosis [42]. Phosphorylation at S19 of PTS serves as a substrate for cGKII phosphorylation and is crucial for its normal activity [43]. This evidence suggests that PKN1, through its downstream substrates, plays a crucial role in biological metabolic processes by modulating enzyme activity via phosphorylation. The co-regulation and functional association of PKN1 phosphosites (S562 and S916) with metabolic enzymes are given in Figure 4.

### 2.8. Co-Regulated Binary Interactors with S562 and S916: Reinforcing the Role in Cytoskeletal Organization and Insulin Signaling

**Figure 5:**
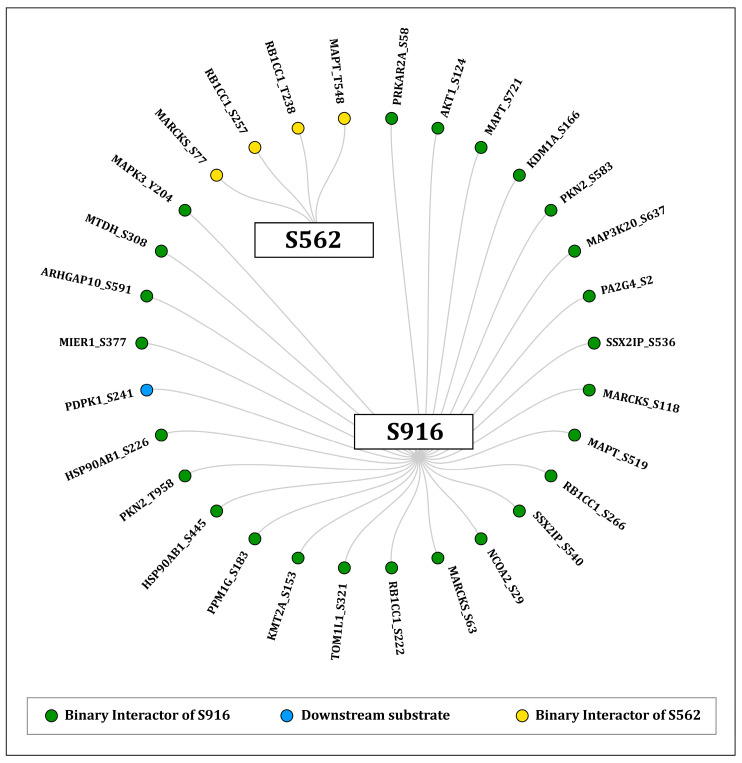
Radial network representation of binary interactors and their co-regulated phosphosites with predominant PKN1 sites S916 and S562.

Among the high-confidence proteins, MARCKS (S77), RB1CC1 (S257, T238), and MAPT (T548) were identified as binary interactors positively co-regulated with S562. MARCKS is a filamentous actin-crosslinking protein that integrates signals from protein kinase C and the calcium (Ca^2+^)/calmodulin pathway to modulate the interactions between the actin cytoskeleton and membrane [44]. MAPT (microtubule-associated protein tau) is crucial for the organization and stabilization of the microtubule cytoskeleton [45]. This aligns with the prediction that S562 is associated with the cytoskeletal regulation of PKN1. In contrast, S916 possesses 26 binary interactors, including downstream substrates PDPK1 (S241) and PKN2 (S583) and upstream kinase MAPK3 (Y204). Notably, PDPK1 phosphorylation at S241 is required for its catalytic activity and activation of downstream signaling [38,39], reinforcing the role of PKN1 S916 within the insulin signaling pathway. A radial network of binary interactors associated with both the S562 and S916 sites is illustrated in Figure 5 and detailed in Supplementary Table S4.

### 2.9. Co-Regulated Phosphorylation of PKN1 and Its Association in Hepatocellular Carcinoma (HCC) Pathogenesis

PKN1 regulates cytoskeletal organization, cell proliferation, and survival through key oncogenic pathways, such as PI3K/AKT/mTOR, TGF-β, and p38-MAPK [46]. Aberrant activation of PKN1 in cancers occurs through overexpression, gene fusions, or deletion of its autoinhibitory N-terminal domain, resulting in constitutive kinase activity. In prostate carcinoma, PKN1 promotes invasion via p38-mediated activation of PXN, NEDD9, and NT5E/CD73, while its inhibition suppresses androgen-dependent transcription [15]. In endometrial and melanoma cells, it modulates TGF-β/EGF and WNT/β-catenin signaling, respectively, thereby influencing tumor proliferation and apoptosis. In glioma, PKN1 overexpression correlates with tumor grade and activates YAP within the Hippo pathway, whereas its inhibition by Raloxifene reduces malignancy and sensitizes cells to Temozolomide [47,48]. Furthermore, PKN1 fusions such as ANXA4-PKN1 and TECR-PKN1 identified in hepatocellular and lung cancers eliminate the N-terminal regulatory domain, driving constitutive activation and uncontrolled proliferation [49]. Collectively, these findings underscore PKN1 as a pivotal oncogenic kinase whose dysregulation promotes tumor growth, invasion, and metabolic adaptation across diverse malignancies.

Hepatocellular carcinoma (HCC) is characterized by activation of multiple cellular signaling pathways, including insulin/IGF signaling [50]. Given that the co-regulated proteins were enriched in insulin signaling and metabolic pathways, we examined these proteins for their association with HCC. To explore gene expression patterns in HCC, we utilized the HCCDB, a curated database of HCC transcriptomes that integrates 15 public datasets covering approximately 4000 samples [51] (http://lifeome.net/database/hccdb, accessed on 23 December 2025). Among these, PKN1 was markedly up-regulated in HCC and positively co-regulated with its histological grading, i67 expression, and Microvessel Density (MVD) [18].

**Figure 6:**
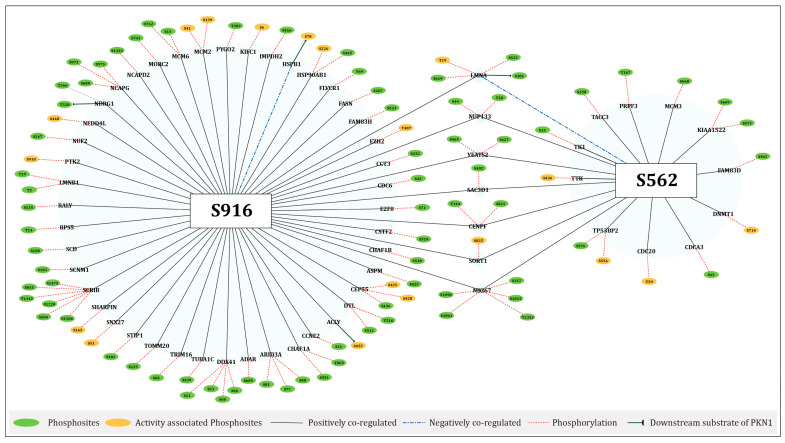
Network representation of overexpressed proteins in hepatocellular carcinoma (HCC) and their overlap with the FET-derived datasets, illustrating proteins and corresponding phosphosites co-regulated with the predominant PKN1 sites S562 and S916. Green nodes denote phosphosites, and orange nodes denote activity-associated phosphosites. Edges represent regulatory relationships: solid black lines indicate positive co-regulation and dashed blue lines indicate negative co-regulation with predominant sites, dashed red lines represent phosphorylation events of specific proteins, and solid green lines indicate the downstream substrates of PKN1. Two central nodes, S916 and S562, display their co-regulated phosphosites in other proteins, highlighting potential kinase–substrate interactions and co-regulation events with proteins associated with HCC.

The high-confidence proteins from our curated datasets with PKN1 predominant sites were subsequently compared with these proteins. In HCC, 50 proteins with 73 phosphosites positively co-regulated with S916, whereas HSPB1 (S78) negatively co-regulated with S916. Similarly, 17 proteins with 21 phosphosites were positively co-regulated with S562, and LMNA (S423, S301) was negatively co-regulated with S562, with all identified proteins showing as up-regulated in HCC. Notably, 15 and 5 POp co-regulated with S916 and S562, respectively, and were classified as activity-associated phosphosites. Protein phosphosite co-regulation networks of S916 and S562 with up-regulated proteins in hepatocellular carcinoma (HCC) are demonstrated in Figure 6.

Among the co-regulated POp, MCM2, an essential DNA replication factor, was significantly overexpressed in HCC tissues relative to normal liver tissues, contributing to tumor progression through promoting the stemness properties of HCC cells [52]. Three phosphosites of MCM2 (S41, S139) are positively co-regulated with S916, where these phosphorylation events have been previously linked to the initiation of DNA replication in eukaryotes [53]. Similarly, CEP55, a regulator of mitotic exit and cytokinesis, was abnormally up-regulated in HCC tissue and acts as an oncogene by promoting the HCC cell migration [54]. Two activity-associated phosphosites of CEP55 (S425, S428, S436), which are essential for its proper midbody localization during cytokinesis [55], were positively co-regulated with S916. Additionally, PTK2, a focal adhesion kinase that activates the cancer stem cell property and promotes tumorigenicity in HCC cells [56], is positively co-regulated with S916. Phosphorylation of PTK2 at S910 represents a crucial regulatory mechanism in focal adhesion disassembly [57].

Collectively, these findings suggest that phosphorylation of PKN1 at S916 and S562 is associated with the pathogenesis of HCC. Proteins co-regulated with S916 are found to be predominantly associated with insulin signaling and metabolic processes, highlighting a potential mechanistic link between PKN1 and HCC.

## 3. Discussion

This study provides the first systematic compilation of phosphoproteomic datasets for PKN1 and highlights two predominant phosphosites, S562 and S916, with potential regulatory relevance. The identification of S916 within the conserved turn motif (TM) of the AGC group of kinases underscores its role as an activation site, consistent with prior evidence showing reduced kinase activity upon mutation [23]. In contrast, S562 represents a non-conserved, highly phosphorylated residue unique to PKN1 functions, suggesting divergent regulatory functions within the PKN family. The conservation of S916 across PKN1, PKN2, and PKN3 and their evolutionary alignment with activation motifs in PKC and AKT reinforce the concept that phosphorylation within the TM is critical for structural stabilization and kinase activity across the AGC group of kinases [14,23,30,31]. Significantly, S562 is found to be the most frequently phosphorylated site in PKN1, and revealing its regulatory pattern might point to a unique functional role associated with PKN1.

The co-regulated protein network of PKN1 S562 explores the crucial role of PKN1-mediated signaling in cytoskeletal organization and cellular structural stability. The proteins exhibiting the positive co-regulation with S562, including ABI2, MARCKS, RB1CC1 and MAPT, are well recognized for their involvement in actin remodeling, membrane cytoskeletal interactions, and microtubule stabilization [44,45]. PKN1 functions as a downstream effector of the Rho family small GTPases, which are key regulators of cytoskeletal dynamics. These findings suggest that phosphorylation at S562 may contribute as a signaling node integrating the Rho-dependent pathways with downstream targets of actin and microtubule organization [13,34]. This regulatory mechanism is important for several cellular functions such as cell motility, adhesion, and division. The disruption of these functions may possibly indicate that S562-associated signaling may be relevant to pathways implicated with neurodegenerative conditions and cytoskeletal-related disorders. Additionally, further experimental validation and investigations on the functional consequences of S562 phosphorylation, particularly on its effects in protein conformation, kinase activity, and substrate interaction, may provide mechanistic insights and novel strategies for therapeutic modulation of cytoskeletal signaling in both physiological and pathological contexts.

Co-regulation network analysis further revealed that S916 is integrated with the insulin signaling pathway and metabolic regulation. Notably, PDPK1 (S241), GFPT2 (S244), GFPT1 (S261), PPP1R3D (S25), IRS2 (S306, S577), PRKAA1 (S496), and AMPK pathway components show positive coregulation with S916, suggesting the association of PKN1 phosphorylation and its downstream signaling with a regulatory phosphorylation network of insulin signaling through activating and inhibitory events [35,36,38,39,40,41,58]. The positive co-regulation of metabolic enzymes such as PI4KB (S511), GFPT1 (S261), PANK2 (S168, S169), PNPLA2 (S404), MVD (S96), GFPT2 (S244), ACLY (S455), GSS (S415), and PTS (S19) further supports PKN1’s potential role in regulating glucose and lipid metabolism [40,42,43,59]. This co-regulation highlights PKN1 as a potential signaling node influencing the insulin signaling pathway and biological metabolic processes by modulating enzyme activity via phosphorylation. However, targeted experimental validation will be essential to establish the functional relevance of these predicted interactions. The co-regulation pattern of S562 phosphorylation reveals a strong association with processes such as chromatin remodeling, mitotic regulation, and DNA repair, while S916 is associated with insulin signaling, AMPK, and metabolic processes. This suggests that PKN1 may function as a signaling integrator, linking cell cycle progression with metabolic adaptation.

In hepatocellular carcinoma (HCC), PKN1 and its phosphorylation at S916 were markedly up-regulated and correlated with tumor grade, Ki-67, and MVD [18]. Quantitative co-regulation analysis revealed 50 proteins (73 phosphosites) positively associated with S916 and 17 proteins (21 phosphosites) with S562, with most showing up-regulation in HCC; 15 and 5 of these sites, respectively, were classified as activity-associated phosphosites. This suggests that phosphorylation of PKN1 at S916 and S562 is associated with the pathogenesis of HCC.

Altogether, our findings suggest that S562 and S916 are not only the most frequently phosphorylated sites in PKN1 but are also functionally distinct nodes of regulation, representing unique and conserved layers of control. Elucidating the upstream kinases and downstream substrates of these sites will be critical for understanding how PKN1 is associated with mitotic control, cytoskeletal dynamics, and metabolic signaling. Targeted experimental validation, particularly in the context of insulin signaling and metabolic processes, will be essential to establish PKN1 as a potential therapeutic target in cancer and metabolic disorders.

## 4. Materials and Methods

### 4.1. Large-Scale Phosphoproteomic Data Integration for Quantitative Mapping of PKN1 Phosphosites

A reproducible search strategy was employed in PubMed using the keywords “phosphoproteomics” or “phosphoproteome”, while excluding the keywords “Plant” and “Review”, to identify the publicly available phosphoproteomics datasets that report the phosphorylation events related to PKN1. Only datasets derived from human cell lines were included for analysis. Each dataset was manually reviewed to extract information on experimentally detected PKN1 phosphorylation sites. The curated phosphoproteomic datasets were obtained through proteolytic digestion using LysC and/or trypsin and were classified based on their phosphopeptide enrichment methods, such as serine/threonine/tyrosine (STY), serine/threonine (ST), or tyrosine (Y) enrichment. To ensure data quality, high-confidence class 1 sites, defined by a localization probability of 75% or higher or an ambiguity score (A-score) exceeding 13, were retained. Identified PKN1 phosphosites were ranked by their frequency of detection across profiling and differential regulation datasets [60].

The curated phosphoproteomics datasets were classified into two categories: profiling datasets, in which experimental and control conditions were treated as independent datasets, and differential datasets, which compared experimental conditions against their corresponding controls. Phosphosites with an absolute fold change greater than 1.3 were defined as upregulated, while those with fold changes below 0.76 were defined as downregulated, based on study-specific statistical significance thresholds (*p* < 0.05) [61].

Proteins identified in each dataset were mapped to their corresponding gene symbols in accordance with the HUGO Gene Nomenclature Committee (HGNC) guidelines. To ensure the uniform annotation across datasets, each phosphosite was linked to the corresponding UniProt accession version using an in-house mapping pipeline [62].

### 4.2. Identification of Frequent and Conserved Predominant Phosphosites of PKN1 from Phosphoproteomics Datasets

Phosphosites classified as class 1 for PKN1 were identified from the integrated datasets and advanced for further evaluation. Phosphosites observed in at least 50% of the profiling and differential datasets were included in subsequent downstream analysis [63]. The sites were ranked according to their recurrence and detection frequency across the compiled datasets. The phosphorylation event that exhibited the highest frequency is defined as the predominant phosphosite of PKN1. Phosphorylation events reported solely from phospho-specific antibody-based studies or mutation-centric experiments, which lacked consistent detection or class 1 classification in large-scale phosphoproteomics datasets, were deliberately excluded [64].

### 4.3. Identification and Filtering of the Co-Differentially Regulated Phosphosites in Other Proteins (POp) as Compared to Predominant Phosphosite-Harboring Phosphosites of PKN1

Phosphosites in other proteins exhibiting positive or negative co-regulation with predominant PKN1 phosphosites were classified according to their co-regulation pattern across differential datasets. PKN1 phosphorylation sites were then systematically paired with phosphosites from other proteins to delineate their differential co-regulation patterns across studies. Each co-regulation pattern was denoted using paired category codes (UU, DD, UD, DU) to describe the regulatory behavior of two components. In each code, the first position represents the regulation status of PKN1, while the second corresponds to the regulation status of phosphosites from other proteins (POp) that were differentially co-regulated with PKN1, with “U” indicating up-regulation and “D” indicating down-regulation. Phosphosites from other proteins that were positively co-differentially regulated with PKN1 were classified as UUDD, whereas those that were negatively co-differentially regulated were categorized as UDDU.

Subsequently, Fisher’s exact test (FET) was applied by generating a contingency table that depicts the association between corresponding PKN1 and other protein sites.

Fisher’s exact test (FET):

$$\sum P=\;\frac{\left(a+b\right)!\left(c+d\right)!\left(a+c\right)!\left(b+d\right)!}{n!}\;\sum_{i}\frac{1}{a_{i}!b_{i}!c_{i}!d_{i}!}$$

Within this analytical framework, “a” denotes the experimental conditions where neither phosphosite was detected, “b” represents the conditions in which only one of the two sites was detected (irrespective of up or down regulation), “c” corresponds to negative co-regulation between the paired sites, and “d” indicates conditions showing positive co-regulation between the paired sites. Fisher’s exact test was applied to each contingency table to assess the statistical significance of co-regulation patterns. Phosphosite pairs with an FET *p*-value below 0.05 in either the positively co-regulated (UUDD) or negatively co-regulated (UDDU) category were considered for further analysis [65].

To minimize potential biases arising from uneven study representation or recurrent sampling of similar experimental settings, an additional stringency filter was implemented. Phosphosite pairs meeting the FET significance threshold were further evaluated using the ratios n(UUDD)/n(UDDU) for positively co-regulated pairs and n(UDDU)/n(UUDD) for negatively co-regulated pairs. Only pairs accounting for at least 10% of the total detection frequency of the corresponding predominant PKN1 phosphosite were retained [66]. These high-confidence PKN1 phosphosites and their co-differentially regulated partners were subsequently subjected to downstream analyses to identify associated biological processes, protein–protein interactions, and kinase–substrate relationships.

### 4.4. Mapping Protein- and Phosphosite-Specific Interactors of PKN1

To comprehensively map the interaction landscape of PKN1, both experimentally confirmed and computationally inferred interactors were gathered from multiple high-quality curated databases. Information on binary and complex protein–protein interactors of PKN1 were obtained from the Biomolecular Interaction Network Database (BIND) [67], CORUM (accessed on 3 March 2023) [68], the Human Protein Reference Database (HPRD) [69], NetPath [70], ConsensusPathDb release 35 (accessed on 22 May 2023) [71], BioGRID [72], and RegPhos 2.0 (accessed on 24 May 2023) [73].

### 4.5. Identification of the Known and Predicted Kinases and Downstream Substrates of PKN1

To investigate the upstream and downstream landscape of PKN1, we performed ki-nase prediction and annotation for its phosphosites using complementary computational and experimental resources. The potential upstream kinases associated with phosphosites of PKN1 were obtained from multiple bioinformatic resources, including NetworKIN (accessed on 4 January 2023) [74] and AKID (accessed on 24 May 2023) [75]. The experimentally validated kinase–substrate associations were obtained from iKiP-DB [76]. Additionally, known PKN1 kinases were also integrated from Johnson et al. (2013) with a cutoff of 90% and above [32].

### 4.6. Approaches to Data Visualization

The lollipop plots illustrating the phosphorylation pattern of PKN1 phosphosites were generated using the AR/Bioconductor package trackViewer (v1.47.0) [77]. The cumulative distribution of phosphosites across the differential datasets was visualized in Python (v3.13.0) using the Matplotlib (v3.10.0) and Pandas libraries (v2.3.3) [78,79]. Clustal Omega (v1.2.4) was used to perform multiple sequence alignment (MSA) [80], and the evolutionary conservation of the predominant phosphosites across and within the kinase families was evaluated with PxKD [24].

Additional visualization and pathway mapping were carried out using RAW Graph 2.0 [81], Cytoscape (v3.10.4) [82], PathVisio 3 [83], Adobe Illustrator (2020), and BioRender (2023) [84].

## 5. Conclusions

Our integrative large-scale analysis of the phosphoproteomics datasets establishes S562 and S916 as the predominant phosphorylation sites of PKN1. S916, embedded within the conserved turn motif (TM) of the AGC group of kinases, emerges as a canonical regulatory site essential for kinase stability and activity. Its strong evolutionary conservation and enrichment across PKN family activation domains further underscore its central role in kinase regulation. In contrast, S562 represents a non-conserved, PKN1-specific phosphorylation site whose high frequency and apparent specificity suggest a unique functional determinant with potential specialization distinct from other AGC group of kinases. Co-regulatory network analysis revealed that phosphorylation of both residues is consistently associated with proteins involved in cell cycle progression, DNA repair, intracellular signaling pathways, and metabolic regulation. Notably, S916 showed particularly strong connectivity to insulin signaling and metabolic pathways, consistent with its conserved role in integrating growth and metabolic cues. Conversely, S562 may function as a selective regulatory switch that fine-tunes PKN1-specific signaling output. Further co-regulation analyses indicate that both phosphorylation events contribute to HCC pathogenesis. Collectively, these findings position PKN1 as a central signaling hub at the interface of cell cycle regulation, DNA damage response, metabolic adaptation, and growth signaling. The identification of S562 and S916 as distinct yet complementary regulatory phosphosites provides mechanistic insight into PKN1 activation dynamics and highlights them as promising candidates for targeted functional studies and therapeutic intervention.

### Limitations of the Study and Future Perspectives

This study significantly enhances the comprehension of the phosphorylational landscape of PKN1 dynamics by incorporating extensive phosphoproteomic datasets; however, several limitations must be acknowledged. The reliance on publicly accessible mass spectrometry data leads to unavoidable heterogeneity stemming from variations in experimental design, sample composition, and data quality among independent studies. Although strict inclusion and filtering criteria were used to prioritize the co-regulated high-confidence phosphosites, the fact that there was no direct biochemical or cellular validation makes the functional interpretation less reliable. Despite these limitations, the identification of S562 and S916 as key regulatory phosphosites provides new insights into PKN1 activation mechanisms. However, future experimental investigations will be required to validate these observations, clarify their mechanistic roles, and evaluate their significance as potential therapeutic targets.

## Data Availability

The original contributions presented in this study are included in the article/Supplementary Materials. Further inquiries can be directed to the corresponding authors.

## Supplementary Materials

The following supporting information can be downloaded at: https://www.mdpi.com/article/10.3390/ijms27052137/s1.
